# Dissecting the mechanisms underlying the accumulation of mitochondrial DNA deletions in human skeletal muscle

**DOI:** 10.1093/hmg/ddu176

**Published:** 2014-04-15

**Authors:** Georgia Campbell, Kim J. Krishnan, Marcus Deschauer, Robert W. Taylor, Doug M. Turnbull

**Affiliations:** 1 Wellcome Trust Centre for Mitochondrial Research, and; 2 Newcastle University Centre for Brain Ageing and Vitality, Institute for Ageing and Health, Newcastle University, Newcastle upon Tyne, UK; 3 Department of Neurology, Martin-Luther-University Halle-Wittenberg, Ernst-Grube Str. 40, Halle (Saale) D-06120, Germany

## Abstract

Large-scale mitochondrial DNA (mtDNA) deletions are an important cause of mitochondrial disease, while somatic mtDNA deletions cause focal respiratory chain deficiency associated with ageing and neurodegenerative disorders. As mtDNA deletions only cause cellular pathology at high levels of mtDNA heteroplasmy, an mtDNA deletion must accumulate to levels which can result in biochemical dysfunction—a process known as clonal expansion. A number of hypotheses have been proposed for clonal expansion of mtDNA deletions, including a replicative advantage for deleted mitochondrial genomes inferred by their smaller size—implying that the largest mtDNA deletions would also display a replicative advantage over smaller mtDNA deletions. We proposed that in muscle fibres from patients with mtDNA maintenance disorders, which lead to the accumulation of multiple mtDNA deletions, we would observe the largest mtDNA deletions spreading the furthest longitudinally through individual muscle fibres by means of a greater rate of clonal expansion. We characterized mtDNA deletions in patients with mtDNA maintenance disorders from a range of ‘large’ and ‘small’ cytochrome *c* oxidase (COX)-deficient regions in skeletal muscle fibres. We measured the size of clonally expanded deletions in 62 small and 60 large individual COX-deficient f regions. No significant difference was observed in individual patients or in the total dataset (small fibre regions mean 6.59 kb—large fibre regions mean 6.51 kb). Thus no difference existed in the rate of clonal expansion throughout muscle fibres between mtDNA deletions of different sizes; smaller mitochondrial genomes therefore do not appear to have an inherent replicative advantage in human muscle.

## INTRODUCTION

Large-scale mitochondrial DNA (mtDNA) deletions are an important cause of mitochondrial disease (1). In many patients these are sporadic single large-scale mtDNA deletions, with the deletion present from birth and the same in all tissues. In other patients, there is a defect of one of the nuclear-encoded maintenance genes and multiple mtDNA deletions develop during life in post-mitotic tissues such as muscle, heart and brain. In patients with mtDNA maintenance disorders the mtDNA deletions found even in adjacent cells is different. This is very similar to the mtDNA deletions which are implicated in the focal cellular dysfunction observed in both ageing and age-related neurodegenerative disease (2–5). Whatever the origin of the large-scale single mtDNA deletion, low levels of mtDNA deletions can be tolerated within a cell due to the multi-copy nature of mtDNA, as a result of the compensatory effects of wild-type mtDNA molecules. An oxidative phosphorylation (OXPHOS) defect, often demonstrated by focal deficiency of cytochrome *c* oxidase (COX) activity, will occur only when the mtDNA deletion load accumulates to exceed a critical threshold level (6). The process by which a single mtDNA species accumulates to predominate within a single cell is known as clonal expansion (7,8). Understanding the mechanism by which this occurs is therefore of paramount importance if we are to gain insight into the progression of mitochondrial disease and the importance of mtDNA deletions in ageing.

To date, three main hypotheses have been proposed for clonal expansion of mtDNA mutations. Clonal expansion was initially hypothesized to be driven by a selective advantage for deleted mtDNA species over wild-type mtDNA molecules based upon replicative turnover (9). This replicative advantage mechanism suggests that the smaller deleted mtDNA molecules are replicated more quickly, permitting mtDNA deletion accumulation (10). An alternative model based on a replicative advantage for mtDNA deletions was proposed some years later, suggesting that the decrease in respiratory chain function associated with mtDNA damage leads to reduced free radical production, resulting in slower turnover of dysfunctional mitochondria and a subsequent accumulation of mtDNA deletions (11). More recently, it has been proposed that a selective advantage is not required for the clonal expansion of mtDNA deletions and that random genetic drift during mtDNA replication is sufficient to allow clonal expansion of a single mtDNA mutation within a cell (12–14) due to relaxed mtDNA replication (15). Random genetic drift is supported by mathematical models based on previously demonstrated principles of mitochondrial replicative dynamics (16). However, the observation of high levels of mtDNA deletions in the majority of substantia nigra neurons from aged individuals (4,5,17) makes this difficult to understand when modelling predicts a low level of age-related COX-deficiency (a maximum of 4% of post-mitotic cells predicted to become COX-deficient by 80 years of age).

Until recently, the presence of a replicative advantage for smaller mtDNA genomes had been considered unlikely based on evidence that some pathogenic mtDNA point mutations, which have no effect on genome size, can accumulate in post-mitotic tissues over time (18). Furthermore, earlier studies of replicative dynamics have shown that mtDNA molecules replicate in 1–2 h (19); as this is significantly less than the half-life of mtDNA—estimated at 1–3 weeks (20,21)—mtDNA replication is unlikely to act as a rate-limiting step for mitochondrial turnover. However, a recent study utilizing a mouse model expressing inducible mitochondria-targeted restriction endonuclease *Pst1* (*mito-Pst1*) presented evidence of a preferential accumulation of genomes harbouring larger mtDNA deletions, renewing interest in the possibility of a replicative advantage for larger mtDNA deletions driving clonal expansion (22).

**Figure 1.**
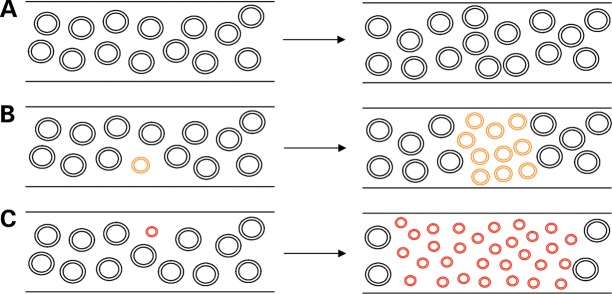
Proposed mechanism for accumulation of smaller mtDNA molecules longitudinally through muscle fibres. Illustration of increased longitudinal accumulation of larger mtDNA deletions through muscle fibres, as predicted by the ‘survival of the smallest’ hypothesis (9). (A) In the presence of no mtDNA deletions, wild-type mtDNA copy number is maintained throughout a muscle fibre over time. (**B**) Following mtDNA deletion formation a replicative advantage over wild-type mtDNA, due to the smaller size of the deleted molecule, causes an increase in the mtDNA deletion level and a spread of the deleted species through the muscle fibre. (**C**) The larger the size of the mtDNA deletion, the smaller the remaining genome and the greater the replicative advantage over wild-type mtDNA. A larger mtDNA deletion size therefore leads to a faster accumulation of the deleted species over the same time span and a greater spread through a muscle fibre .

We wished to test the presence of a replicative advantage for smaller mtDNA molecules in human tissue. The investigation of skeletal muscle from patients with Mendelian-driven disturbance of mtDNA maintenance offers an opportunity to test this hypothesis. Muscle from these patients contains many different deletions of different sizes, with each affected muscle fibre possessing a different, clonally expanded mtDNA deletion causing COX-deficiency (23). The COX-deficient segment has been shown to be of variable length, ranging from <10 to >1000 μm (24). These are thought to occur as a consequence of the continuous replication of mtDNA in muscle cells, associated with mitochondrial fission and fusion, leading to changes in the proportion of mutant mtDNA in muscle segments. This would eventually propagate down the length of a fibre through the mixing of adjacent mitochondria and mitochondrial genomes. Since each COX-deficient segment represents a different, clonal expansion event, we hypothesized that if smaller mitochondrial genomes replicate faster than larger genomes, COX-deficient segments would be larger in the presence of the smallest mtDNA molecule (Fig. 1). Thus by comparing large, COX-deficient areas with smaller COX-deficient areas from the same patient, we hoped to provide new insights into the mechanism of clonal expansion in a relevant human tissue.

## RESULTS

### Assessing COX-deficient muscle fibre region length and area

**Figure 2.**
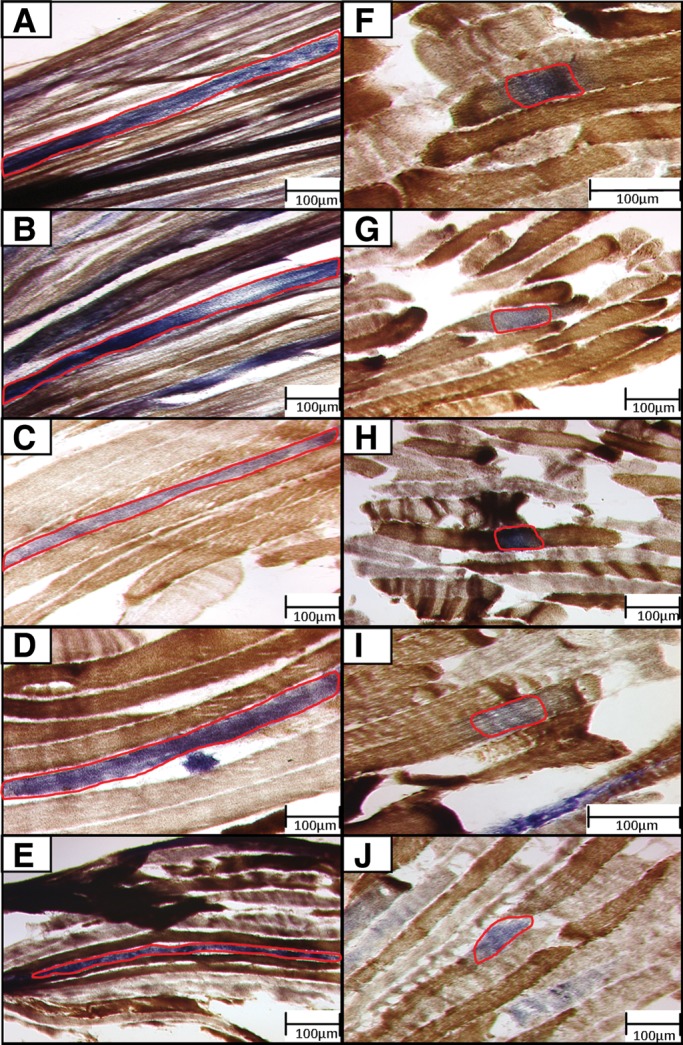
Images of ‘large’ and ‘small’ COX-deficient fibre regions selected for extraction by laser microdissection. Panel of 10 images of longitudinal muscle sections examined using COX/SDH histochemistry, captured prior to laser microdissection—these are representative of typical cutting areas. (**A–E**) Depict five large COX-deficient fibre regions, where the outlined region of COX-deficiency exceeds 500 µm in length. (**F**–**J**) Depict five small COX-deficient fibre regions, where the outlined region of COX-deficiency remains under 200 µm in length. All LMD cutting areas for single fibre isolation are outlined in red.

**Figure 3.**
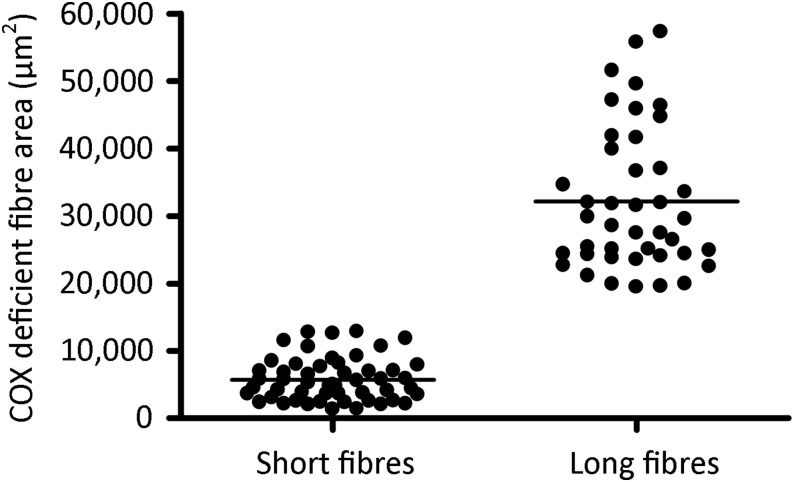
Assessment of COX-deficient fibres captured by laser microdissection. Scatter plot of all captured COX-deficient fibre areas (µm^2^), as measured by the LMD system. Captured fibres were grouped into two types; short (under 200 µm in length) and large (over 500 µm in length). Captured area was assessed to ensure no overlap existed in terms of size between these two groups. Small COX-deficient fibres: mean COX-deficient fibre area = 5725 ± 3194 µm^2^. Large COX-deficient fibres: mean COX-deficient fibre area = 32 120 ± 3194 µm^2^. The two groups of COX-deficient muscle fibre areas were found to be significantly different using a Mann–Whitney test (*P* < 0.0001).

Longitudinal sections of muscle biopsy underwent histochemical assessment of COX activity; COX-deficient regions of muscle fibres were targeted for investigation in this study, representing fibre areas where mitochondrial activity has been disrupted to a sufficient degree by mtDNA deletion accumulation to cause a biochemical defect. Two categories of fibre were selected for investigation; ‘large’ (over 500 μm in length) and ‘small’ (under 200 μm in length) regions of COX-deficiency (Fig. 2). Values were chosen based on a maximum cutting length on the microscope/LMD system of 650 μm. The area (µm^2^) of single muscle fibres isolated by laser microdissection was determined at the time of cutting and was found to be a more variable measurement than fibre length when extracting fibres. However, no overlap was observed between the two groups in terms of COX-deficient fibre area (*P* < 0.0001, Mann–Whitney test); small COX-deficient fibre regions had a mean area of 5725 ± 3194 µm^2^ (range 1302–12 800 µm^2^) compared with 32 120 ± 10 536 µm^2^ (range 19 412–57 289 µm^2^) for large COX-deficient regions (Fig. 3A). Although large and small COX-deficient muscle fibre regions were selected based upon the length of the fibre region affected by the biochemical defect, we decided that total fibre area (µm^2^) isolated by laser microdissection would be a more appropriate and reliable measure. The area of COX-deficiency is a better measure of the spread and accumulation of the mtDNA deletion species responsible for the biochemical defect since measurements based on length fail to take into consideration fibre-width variation.

### Characterizing mtDNA deletion size in single COX-deficient muscle fibre regions

A total of 156 COX-deficient muscle fibre regions were successfully amplified by long-range PCR; from this group, a single PCR amplimer representing one individual clonally expanded mtDNA deletion was observed in 122 muscle fibres. These corresponded to 62 small (under 200 μm in length) COX-deficient fibres and 60 large (over 500 μm in length) COX-deficient fibres which were included in the final analysis of mtDNA deletion sizes. Amplimer sizes from these fibre groups were assessed by gel electrophoresis, with comparison to a 1 kb DNA ladder allowing an estimate of mtDNA deletion sizes to be obtained (Fig. 3B). Multiple mtDNA deletions were detected in the remaining 34 COX-deficient fibre regions which were successfully amplified, with an even distribution of large (15 total) and small (19 total) COX-deficient fibre regions. These fibres were not included in the final analysis since it was not possible to establish how large a region of COX-deficiency either of the mtDNA deletion species is responsible for.

To ensure that mtDNA deletion size estimates from gel electrophoresis PCR bands were a true reflection of mtDNA deletion size, a stratified sample of 20 single fibres (10 large and 10 small COX-deficient fibre regions) were selected for further analysis by breakpoint sequencing. All sequenced mtDNA deletion breakpoints are consistent with the mtDNA deletion size predicted by gel electrophoresis (mean deviation of 229 ± 191 bp), demonstrating that long-range PCR can be used to accurately assess mtDNA deletion size (Table 1).

**Table 1.** mtDNA deletion sizes in COX-deficient fibre regions as determined by PCR/gel electrophoresis and mtDNA breakpoint sequencing

COX- deficient fibre	Estimated mtDNA deletion length (kb)	Verified mtDNA deletion length (bp)	Size difference (bp)	5′ Deletion breakpoint	5′ Breakpoint sequence	3′ Deletion breakpoint	3′ Breakpoint sequence	Breakpoint repeat type
1	3.5	3715	215	9576	***a***tc***a***c***c***ccgc	13 291	***a***ca***a***t***c***ggca	No repeat
2	3.5	3904	404	10 265	tctagaaatt	14 269	atcctcccga	No repeat
3	5	4977	23	8482	***tccctcacca***	13 459	***tccctcacca***	***Direct repeat***
4	6.5	6994	444	9075	***aa***tatcaac***c***	16 069	***aa***gtattga***c***	No repeat
5	7	7479	479	7939	***a***ctaat***c***t***tc***	15 436	***a***gacgc***c***c***tc***	Imperfect repeat (***4/10***)
6	7.5	7648	148	6341	aga***ccta***ac***c***	13 989	gcc***ccta***ct***c***	Imperfect repeat (***5/10***)
7	8.5	8493	7	5438	***a***aaacccacc	13 931	***a***ccctagcat	No repeat
8	9	9033	33	6558	a***a***cac***c***a***cc***t	15 591	c***a***att***c***t***cc***g	Imperfect repeat (***4/10***)
9	9	9026	26	6634	***c***tga***a***g***t***tta	15 660	***c***aat***a***a***t***ccc	No repeat
10	9	9120	120	6468	tg***a***tccg***tc***c	15 588	ac***a***caat***tc***t	No repeat
11	2.5	2880	120	7236	gac***tacc***c***c***g	9516	ttt***tacc***a***c***t	Imperfect repeat (***5/10***)
12	5	4977	23	8482	***tccctcacca***	13 459	***tccctcacca***	***Direct repeat***
13	6	5647	353	7406	***c***ccccc***a***cc***c***	13 053	***c***ataga***a***gg***c***	No repeat
14	6.5	6175	325	7947	***t***caa***c***t***c***ct***a***	14 122	***t***tcc***c***a***c***tc***a***	Imperfect repeat (***4/10***)
15	7.5	7046	464	5787	***a***a***gc***tgctt***c***	12 833	***a***t***gc***caaca***c***	Imperfect repeat (***4/10***)
16	9.5	9044	456	6105	a***t***aa***tc***ttc***t***	15 149	g***t***cc***tc***ccg***t***	Imperfect repeat (***4/10***)
17	10	9846	154	5341	tta***a***cc***t***ct***a***	15 187	agt***a***at***t***ac***a***	No repeat
18	6.5	6927	427	8734	***a***cctgat***c***t***c***	15 661	***a***ataatc***c***c***c***	No repeat
19	8	8026	26	7634	tcccctat***c***a	15 660	caataatc***c***c	No repeat
20	3	3250	250	12 140	t***c***ct***c***ttg***t***a	15 390	c***c***tc***c***cat***t***c	No repeat

COX-deficient fibres 1–10 were small COX-deficient fibres, while 11–20 were large COX-deficient fibres. Estimated mtDNA deletion length refers to mtDNA deletion size as measured to the nearest 0.5 kb by long-range PCR and gel electrophoresis, while verified mtDNA deletion size refers to the deletion size ascertained by breakpoint sequencing; size difference refers to any discrepancy between the estimated and verified mtDNA deletion sizes in base-pairs. mtDNA sequences of 10 bp are supplied 5′ and 3′ of the mtDNA deletion, with repeated bases in bold and italicized. Two direct repeats (identical flanking sequences) and seven imperfect repeats (4–9 bp repeated between flanking sequences) were identified.

### No difference in mtDNA deletion size observed in large and small COX-deficient muscle fibre areas

**Figure 4.**
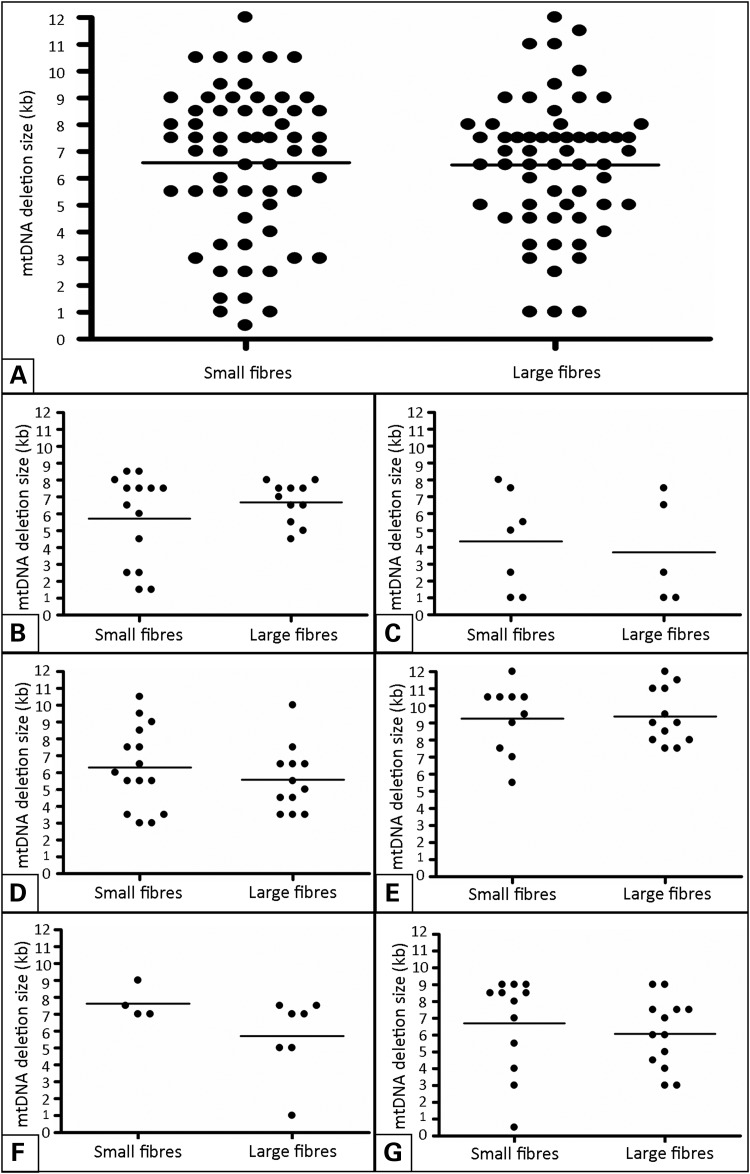
mtDNA deletion sizes present in (**A**) the total dataset, and (**B–G**) datasets from each patient. Assessment of mtDNA deletion sizes present in large and small COX-deficient fibres, depicted by scatter plot and carried out by Mann–Whitney *U* test (non-Gaussian data distribution) or unpaired *t*-test (normally distributed data). (A) Small COX-deficient fibres, mean mtDNA deletion size = 6589 bp (±2788 bp, *N* = 62); large COX-deficient fibres, mean mtDNA deletion size = 6508 bp (±2478 bp, *N* = 60). No significant difference was found to exist between the two fibre groups (*P* = 0.5506), with a similar range of mtDNA deletion sizes displayed in both. Similar results are seen for each of the six patient datasets (B–G), with no significant difference in deletion size between the two fibre groups in any single case (*P* = 0.7009, 0.7751, 0.4136, 0.8272, 2303 and 0.3406, respectively).

Analysis of the 60 large COX-deficient fibre regions showed a range in mtDNA deletion size from 1 to 11.5 kb, with a mean mtDNA deletion size of 6.51 ± 2.32 kb (mean ± SD). In the 62 small COX-deficient fibres, mtDNA deletion size ranged between 0.5 and 10.5 kb, with a mean mtDNA deletion size of 6.59 ± 2.07 kb (mean ± SD) (Fig. 4A). The use of a 10 kb long-range PCR for analysis of a significant proportion of fibres may have caused a slight skew towards smaller deletion sizes and decreased the average identified mtDNA deletion size; however, the two PCR assays were used in a similar ratio across the two data sets, and therefore should have no impact on further data analysis. As the large and small fibre data were not found to follow a normal distribution, a Mann–Whitney test was used to compare the two datasets; this identified no significant difference exists between mtDNA deletion sizes found in large and in small COX-deficient muscle fibre areas (*P* = 0.5506). To ensure that no trends linking mtDNA deletion size and affected COX-deficient muscle fibre area in individual patients are being masked by inclusion into the total dataset, data from individual cases were also analyzed independently (Fig. 4B–G). No significant difference between mtDNA deletion sizes in large and in small COX-deficient muscle fibre areas was identified in any of the six cases included in this study (Table 2) in keeping with the total dataset.

**Table 2.** Average deletion sizes of ‘large’ and ‘small’ COX-deficient fibres in six individual multiple mtDNA deletion patients.

Patient	Small fibre, average deletion size (kb)	Large fibre, average deletion size (kb)	Difference of deletion size in large fibres compared with small fibers (kb)
1	*N* = 14, 5.71 ± 2.66	*N* = 11, 6.68 ± 1.21	+0.97, *P* = 0.7009
2	*N* = 7, 4.36 ± 2.30	*N* = 5, 3.70 ± 3.09	−0.66, *P* = 0.7751
3	*N* = 15, 6.30 ± 2.43	*N* = 12, 5.58 ± 1.94	−0.72, *P* = 0.4136
4	*N* = 10, 9.25 ± 2.02	*N* = 12, 9.34 ± 1.61	+0.13, *P* = 0.8727
5	*N* = 4, 7.63 ± 0.97	*N* = 7, 5.71 ± 2.34	−1.91, *P* = 0.2303
6	*N* = 12, 6.71 ± 2.83	*N* = 13, 6.08 ± 2.06	−0.63, *P* = 0.3406
Total	*N* = 62, 6.59 ± 2.79	*N* = 60, 6.51±2.48	−0.08, *P* = 0.5506

No trend was observed across the six patient biopsies investigated for large mtDNA deletions to associate with either small or large regions of COX-deficiency in muscle fibres.

### Assessing the presence of a correlation between mtDNA deletion size and COX-deficient skeletal muscle fibre area

**Figure 5.**
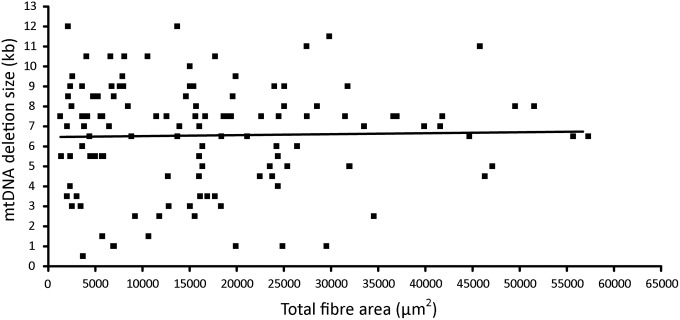
Correlation between mtDNA deletion size and COX-deficient fibre area. Spearman's correlation analysis carried out on the total data set of 122 COX-deficient skeletal muscle fibre regions, to determine if any relationship exists between mtDNA deletion size and the size of the resulting area of biochemical deficiency. Relationship between these variables depicted here by scatter plot and linear regression line. No significant correlation was found to exist between these two parameters (*P* = 0.7792).

To ensure that we were not missing a trend, correlation analysis of mtDNA deletion size and COX-deficient fibre area in the complete dataset of 122 individual fibres was undertaken. No significant correlation was found to exist between mtDNA deletion size and COX-deficient skeletal muscle fibre area of the 122 biochemically deficient fibre regions successfully analyzed (*P* = 0.7792, Spearman correlation analysis) (Fig. 5). Separate analyses of the small and the large COX-deficient fibre region datasets similarly revealed no significant correlation between mtDNA deletion size and area (µm^2^) of skeletal muscle fibre COX defect (*P* = 0.7140 and 0.7871, respectively).

As for the assessment of differences in mtDNA deletion size between large and small COX-deficient skeletal muscle fibre areas, data sets from each patient were individually analyzed to preclude any trend in a single patient becoming obscured when included within the total dataset. No significant correlation was found to link mtDNA deletion size to the size of the area of COX-deficiency in muscle fibres in any of the six investigated cases (*P* = 0.2477, 0.8572, 0.2477, 0.4438, 0.4590 and 0.8599, respectively).

## DISCUSSION

This study aimed to test the hypothesis that the clonal expansion of large-scale mtDNA deletions, leading to focal cellular biochemical defects in post-mitotic tissues in patients with mitochondrial disease and in ageing, is driven by a replicative advantage for mtDNA deletions over wild-type mtDNA due to a smaller genome size. We proposed that if mtDNA deletion species have a replicative advantage over wild-type, based on a smaller size and faster replication time, larger mtDNA deletions would display the same replicative advantage over smaller mtDNA deletions. In this case, we would observe the largest mtDNA deletions accumulating at the greatest rate, and hence spreading furthest longitudinally through skeletal muscle fibres. However, in this study we see no significant difference in size of mtDNA deletion between laser microdissected large and small COX-deficient fibre areas from patients with mtDNA maintenance disorders leading to COX-deficiency.

Interestingly, our study arrives at different conclusions to the elegant studies of Moraes and colleagues who have attempted to investigate the mechanism in different model systems. Using *trans*mitochondrial cybrids derived from patient fibroblasts with high heteroplasmy levels of single, large-scale mtDNA deletions, these authors demonstrated a replicative advantage for mtDNA deletions over wild-type molecules in the same cell under relaxed mtDNA copy number conditions following ethidium bromide-driven mtDNA depletion (25). In addition, they presented evidence to show that smaller mitochondrial genomes accumulate more rapidly *in vivo* in a mito*Pst1* mouse model, developed to express a neuronal-specific inducible mitochondrial-targeted restriction endonuclease (*Pst1*). The induction of mito*Pst1* causes the generation of double-strand mtDNA breaks, with the subsequent formation of mtDNA deletions closely mimicking the range of naturally occurring mtDNA deletions found in ageing neurons. These authors reported preferential accumulation of genomes harbouring larger mtDNA deletions compared with smaller mtDNA deletions, concluding that this was due to a replicative advantage for smaller mitochondrial genomes (22).

A possible explanation for the discrepancies between our results and the findings from previous studies is that there are times when replication rate does become rate limiting. The data of Diaz *et al.* (25) would support this because given a replicative advantage for mtDNA deletions was only found under relaxed copy number control in the mtDNA deletion cytoplasts. No accumulation of, or selective advantage for, mtDNA deletions was reported under normal copy number control (25), in agreement with our own findings in patient tissue. The rate of mtDNA replication might be very important during the process of mtDNA deletion formation and in establishing which deletions clonally expand, but not the rate of clonal expansion once the deletions have become established. In addition, the clonal expansions we detected were associated with a clear biochemical defect. Under these circumstances the pressure of mitochondrial biogenesis may have overwhelmed any effect of deletion size. This could explain the difference in our findings and those of Moraes *et al.* in the mito*Pst1* mouse, where there was no biochemical defect. We did observe a high prevalence of larger mtDNA deletions in the clonally expanded segments (75% were >5 kb and 50% were >6.5 kb); this is in keeping with the observation of an mtDNA deletion size distribution in COX-deficient fibre regions where 70% were >5 kb and 45% were >6.75 kb in aged muscle fibres in human skeletal muscle (26). However, this could also be due to larger deletions being formed more easily following an initial mutation event.

There are still many uncertainties in our understanding of mitochondrial biology in muscle cells. We have assumed that the size of COX-deficient segments correlates with the rate of clonal expansion but of course other factors would influence this such as the structure of the muscle itself, rate of mitochondrial fission and fusion, and movement of mitochondria in and out of the respiratory deficient region of the fibre. However, if we assume that these would be the same for long and short respiratory deficient segments then the lack of any evident difference in deletion size within an individual does not favour a greater rate of clonal expansion of the larger deletions.

Another challenge when considering our results is the natural history of the respiratory deficient segments. The variation in segment size is well documented but it is not known whether these segments gradually increase in size or are constantly being lost over time. We are not aware of any longitudinal studies that have looked at this in detail, though an accumulation of mtDNA deletions over time has been shown to lead to fibre loss and breakage in a rat model (27). It is therefore possible that the size of the respiratory deficient segment is due to factors other than the rate of clonal expansion, although the evidence of increased mitochondrial biogenesis in the presence of a respiratory chain defect (28) suggests it will have a role.

The importance of clonal expansion in causing cellular pathology in ageing and disease is highlighted by the ability of cells to maintain normal function in the presence of low-level mtDNA deletions; the initial mtDNA deletion formation event would be unlikely to lead to a biochemical defect without the intercession of clonal expansion. It is consequently of paramount importance to understand the mechanism by which clonal expansion occurs in order to prevent the consequences of mitochondrial deficiency in ageing and disease. We have observed no variation in the ability of mtDNA deletions of different sizes to accumulate longitudinally through skeletal muscle fibres, leading us to the conclusion that smaller mtDNA molecules do not possess a replicative advantage to drive forward clonal expansion in human muscle. While we have focussed upon the finding that smaller genome size is not an advantage, it is important to note that we have seen no trend toward a greater rate of clonal expansion for the smallest mtDNA deletions either; this implies that no disadvantage is associated with losing larger sections of the genome through a deletion event. Overall, we have found no evidence to support the presence of a selective pressure based upon mtDNA deletion size to drive clonal expansion once the deletion has formed.

## MATERIALS AND METHODS

### Patients and muscle biopsy samples

Skeletal muscle samples (quadriceps or biceps brachii) were obtained by open muscle biopsy for diagnostic purposes or at post-mortem from six patients with multiple mtDNA deletions detected in muscle by long-range PCR and/or Southern blotting due to a disturbance in mtDNA maintenance; five patients had a genetic diagnosis of recessive POLG mutations, while the underlying molecular genetic defect in a sixth case remains undetermined despite excluding commonly reported nuclear genetic defects. A full summary of the clinical, molecular and histochemical findings in all cases is presented in Table 3. The availability of large biopsy samples in these patients permitted longitudinal sections to be cut for analysis along the length of skeletal muscle fibres. Samples were frozen in isopentane cooled by liquid nitrogen for histological and histocytochemical analysis. Ethical approval for our study was obtained from Newcastle and North Tyneside LREC.

**Table 3.** Clinical, histological and molecular genetic characterization of patients with multiple mtDNA deletions in muscle.

Patient	Age at biopsy	Clinical presentation	Nuclear genetic defect	Histochological abnormalities in muscle	References
1	59	Chronic progressive external ophthalmoplegia (CPEO), myopathy, ataxia and parkinsonism.	p.Gly848Ser and p.Ser1104Cys *POLG* mutations	15% COX-deficient fibres, 5% RRF	Betts-Henderson *et al.* (2009) (29)
2	74	CPEO	p.Thr251Ile/p.Pro587Leu and p.Arg627Gln *POLG* mutations	15–20% COX-deficient fibres, 1% RRF	This study
3	55	SANDO	p.Ala467Thr and p.Trp748Ser *POLG* mutations	7% COX-deficient fibres, 1% RRF	This study
4	50	CPEO, ataxia, parkinsonism and neuropathy	p.A467T and p.X1240Q *POLG* mutations	20% COX-deficient fibres	Cottrell *et al.* (2000) (30); Lax *et al.* (2012) (31)
5	63	CPEO	Multiple mtDNA deletion disorder with no known nuclear genetic defect; mutations in *POLG*, *POLG2*, *PEO1*, *SLC25A4*, *RRM2B* and *TK2* excluded	20% COX-deficient fibres, 5% RRF	This study
6	80	CPEO	p.(Thr251Ile)/p.(Pro587Leu) and p.(Ala467Thr) *POLG* mutations	15% COX-deficient fibres, 3% RRF	This study

COX, cytochrome *c* oxidase; RRF, ragged-red fibres; SANDO, sensory ataxic neuropathy with dysarthria and ophthalmoplegia.

### Mitochondrial enzyme histochemistry

Cryostat sections were cut at a thickness of 20 µm from longitudinally orientated muscle blocks and mounted onto PEN membrane slides (Leica Microsystems) prior to histochemical staining as previously described for the sequential assay of COX/succinate dehydrogenase (SDH) activity (32). Briefly, sections were reacted for 45 min at 37°C with COX reaction media (4 mM diaminobenzidine tetrahydrochloride, 100 μM, cytochrome *c* and 20 μg/ml catalase in 0.2 μM phosphate buffer, pH 7.0) and 40 min at 37°C with SDH media (1.5 mM nitroblue tetrazolium, 1 mM sodium azide, 200 μM phenazine methosulphate, 130 mM sodium succinate, in 0.2M phosphate buffer, pH 7.0).

### Single cell laser microdissection and cell lysis

COX-deficient areas of muscle fibres were isolated by laser microdissection (laser microdissection (LMD) 6000; Leica Microsystems) and collected into the cap of a 0.5 ml microcentrifuge cap (Eppendorf, UK). The size of the area to be cut was measured, using the Leica Microsystems software, and recorded. Isolated COX-deficient muscle fibre regions were lysed in 15 μl of single cell lysis buffer (500 mM Tris–HCl, 1% Tween 20, dH_2_0, 1% proteinase K) and incubated at 55°C for 2 h followed by 95°C for 10 min.

### Long-extension PCR to detect mtDNA deletions

We used long-extension PCR to detect and characterize mtDNA deletions within individual COX-deficient muscle fibres. Over the course of the study, two sets of primer pairs were used; one set amplified a ∼13.5 kb region of the major arc (amplifying between mtDNA nucleotides 2999–16 450; GenBank Accession number: NC_012920.1), whilst a second primer set amplified a ∼10.0 kb region within the same section of the major arc of the mitochondrial genome (nucleotides 6240–16 132). Both sets of data were used—28 fibres were investigated with the 13.5 kb PCR, while the remaining 94 fibres were assessed using the 10 kb assay.

For the PCR reaction, 1 µl cell lysate was added to PCR mastermix (dH_2_O, LA buffer (TaKaRa), 10 mM dNTPs, 20 mM forward and reverse primers and LA Taq enzyme (TaKaRa), to a total volume of 50 μl. DNA was then amplified under cycle conditions: 94°C for 1 min; 35 cycles of 94°C for 30 s, 58°C for 30 s and 68°C for 11 min; final extension of 72°C for 10 min. PCR products were separated through a 0.7% agarose gel with a 1 kb ladder used to size PCR amplimers.

### mtDNA sequencing analysis

Long-range PCR amplimers were directly sequenced using primers close to the predicted mtDNA deletion breakpoint (BigDye Termination Chemistry on ABI 3130xl genetic analyser; Applied Biosystems); the predicted breakpoint location was determined using a restriction digest with four restriction endonucleases (*Xho*I, *BamH*I, *Xcm*I and *Dra*I, New England Biolabs) (33). ABI PRISM SeqScape Software Version 2.6 was used to identify mtDNA deletion breakpoints, and accurately determine the size of the deleted region of mtDNA.

### Statistical analysis

All statistical analysis was carried out using GraphPad Prism v.4 statistical software. Group comparisons were made using unpaired *t-*tests or Mann–Whitney tests, and data correlation was assessed using a two-tailed Spearman or Pearson correlation test at 95% confidence; relevant tests were chosen based upon data distribution (i.e. depending upon whether or not the spread of data followed a normal distribution, in order to maximize the power of each statistical analysis). An a priori power analysis was carried out in order to calculate the minimum sample size required to observe a significant difference in deletion size between ‘large’ and ‘small’ COX-deficient regions. This test was carried out using the highest standard deviation seen within the groups (2.74 kb), demonstrating a need for a minimum of 36 samples per group to observe a difference in mtDNA deletion size of at least 2 kb.

## FUNDING

Funding to pay the Open Access publication charges for this article was provided by Medical Research Council.
